# Inflammatory Biomarkers and Delirium Severity in Critically Ill Adults: A Prospective Single-Center Cohort Study

**DOI:** 10.3390/jcm15156025

**Published:** 2026-08-03

**Authors:** Mateusz Szczupak, Marek Konop, Sabina Krupa-Nurcek

**Affiliations:** 1 Department of Anesthesiology and Intensive Therapy, Nicolaus Copernicus Hospital in Gdańsk, 80-803 Gdańsk, Poland; 2 Department of Otolaryngology, Faculty of Medicine, Medical University of Gdańsk, 80-210 Gdańsk, Poland; 3 Laboratory of the Center for Preclinical Research, Department of Experimental Physiology and Pathophysiology, Medical University of Warsaw, 02-091 Warszawa, Poland; marek.konop@wum.edu.pl; 4 Department of Surgery, Faculty of Medicine, Collegium Medicum, University of Rzeszów, 35-310 Rzeszów, Poland

**Keywords:** delirium, CAM-ICU-7, C-reactive protein, interleukin-6, procalcitonin, intensive care, inflammation

## Abstract

**Background:** This study examined associations between C-reactive protein (CRP), interleukin-6 (IL-6), ordinal procalcitonin (PCT) categories, and delirium severity in critically ill adults. Secondary exploratory analyses evaluated whether baseline CRP and IL-6 discriminated subsequent incident delirium and remained associated with this outcome after adjustment. **Methods:** This prospective observational cohort included 267 adults treated in a general intensive care unit. Delirium severity was assessed using CAM-ICU-7 at 12 h after admission and on days 2, 4, 7, and 14. CRP and IL-6 were analyzed as continuous variables, whereas PCT was analyzed as a three-level ordinal variable across all five assessment times. Associations with delirium severity and intensive care unit length of stay were evaluated using Spearman rank correlations. Among patients without delirium at 12 h, bootstrap receiver operating characteristic analysis and Firth-penalized logistic regression adjusted for age and sex were performed as secondary exploratory analyses. PCT was not included in these prospective analyses because only ordinal PCT categories were available. **Results:** Delirium was recorded at least once in 38 of 267 patients (14.2%). Thirteen patients had delirium at 12 h, and 25 additional patients subsequently developed incident delirium. Concurrent correlations with CAM-ICU-7 severity ranged from 0.271 to 0.898 for CRP and from 0.312 to 0.735 for IL-6. Concurrent correlations for ordinal PCT categories were 0.304, 0.666, 0.711, 0.720, and 0.329 at the five respective assessment times. All concurrent associations were positive and statistically significant at *p* < 0.001. PCT categories also showed weak positive correlations with intensive care unit length of stay, with coefficients ranging from 0.151 to 0.281. Among 254 patients without delirium at 12 h, baseline CRP showed an AUC of 0.969 (95% bootstrap CI, 0.923 to 0.997), compared with an AUC of 0.653 (95% bootstrap CI, 0.569 to 0.750) for IL-6. Both baseline CRP concentrations and incident delirium were concentrated at floor values in most patients, and this discrimination should be read accordingly as a largely binary separation between floored and elevated CRP rather than a graded biomarker-severity gradient. In the joint Firth model, CRP remained associated with incident delirium, with an adjusted odds ratio of 10.75 per 5 mg/L increase, 95% confidence interval 4.50 to 33.18, *p* < 0.001. IL-6 was not associated with incident delirium after adjustment for CRP, age, and sex, with an adjusted odds ratio of 0.81 per 5 pg/mL increase, 95% confidence interval 0.42 to 2.04, *p* = 0.510. **Conclusions:** CRP, IL-6, and ordinal PCT categories were positively associated with concurrent delirium severity, although no consistent biomarker hierarchy was observed across assessment times. In secondary exploratory analyses, baseline CRP showed high apparent within-sample discrimination for subsequent incident delirium and remained associated with the outcome after adjustment for IL-6, age, and sex. However, the cutoff, discrimination, and effect estimates were derived from a single-center cohort with only 25 incident events. Model calibration, optimism-corrected performance, net clinical benefit, and clinical utility were not evaluated. These findings should not be interpreted as establishing a diagnostic test or clinical prediction tool and require validation in independent external cohorts before any clinical application.

## 1. Introduction

Delirium is one of the most common manifestations of acute brain dysfunction in critically ill patients and remains a significant, yet under-recognized clinical problem in intensive care units. Current reviews emphasize that the duration of delirium in ICU patients is independently associated with increased mortality, prolonged hospitalization, increased treatment costs, and poorer long-term cognitive outcomes. Clinically, delirium is not merely a transient disturbance of consciousness, but a marker of disease severity and a factor contributing to short- and long-term prognosis [1,2,3]. The prevalence of delirium in the ICU patient population is estimated to range from 32% to as much as 87%, making it a significant clinical problem in modern intensive care [4,5,6,7].

The etiopathogenesis of delirium is complex and multifactorial. Key mechanisms of its development include disturbances in brain energy metabolism, neurotransmitter dysfunction, and inflammatory processes that damage central nervous system structures. Growing evidence indicates that a systemic inflammatory response plays a significant role in the pathogenesis of delirium, leading to microglial cell activation and increased blood–brain barrier permeability [8,9]. The relationship between markers of systemic inflammation and the occurrence of delirium and coma during critical illness is particularly interesting. A study published in 2024 demonstrated a link between inflammatory markers and the activity of endogenous anticoagulant systems, as well as the occurrence of delirium and coma during critical illness, reinforcing the concept that the inflammatory response is not merely an epiphenomenon of the patient’s serious condition but may actively contribute to the development of acute brain dysfunction. Previous prospective biomarker analyses also indicated that markers of inflammation and astrocytic and glial activation correlate with longer delirium duration and greater symptom severity [10,11]. In this context, readily available biomarkers in clinical practice, such as C-reactive protein, interleukin-6, and procalcitonin, are of particular importance. The most recent systematic review from 2025, encompassing 28 studies and 54 serum biomarkers assessed in critically ill adults, indicated that inflammatory markers, including CRP and IL-6, are among the most frequently analyzed biological indicators of delirium. However, the authors emphasized that although the biological association is plausible, and elevated CRP and IL-6 concentrations have been associated with blood–brain barrier permeability and microglial activation, the results to date remain methodologically heterogeneous and not fully consistent [12]. At the same time, more recent clinical studies suggest that these markers may have predictive value. A study published in 2025 demonstrated that postoperative CRP, IL-6, and procalcitonin levels in ICU patients were positively correlated with both the occurrence and severity of postoperative delirium and, in multivariate analysis, remained independent predictors. These data do not unequivocally establish causality, but they support the hypothesis that an enhanced inflammatory response may facilitate the early identification of patients at particular risk of acute brain dysfunction [13]. However, the available literature warrants caution in interpretation. Some biomarker studies indicate that the significance of individual markers may depend on the patient population, measurement timing, delirium phenotype, and comorbidities such as coma or infection. This heterogeneity in data is one of the most important arguments for conducting further prospective studies with clearly defined time points for laboratory tests and standardized assessment of delirium [9,12].

Despite the growing number of studies demonstrating an association between inflammation and delirium, there remains a lack of clear data to determine the role of specific inflammatory biomarkers in predicting this complication. Of particular interest are markers such as C-reactive protein, interleukin-6, and procalcitonin, which reflect the severity of the systemic inflammatory response and may have prognostic significance in critically ill patients [10,12].

The primary aim of this study was to examine associations between serial inflammatory biomarker measurements and concurrent delirium severity in adults treated in a general ICU. Delirium severity was assessed with CAM-ICU-7 at five prespecified time points. We hypothesized that higher CRP and IL-6 concentrations and higher ordinal PCT categories would be associated with greater concurrent delirium severity. As a secondary exploratory objective, we evaluated the apparent within-sample discrimination of baseline CRP and IL-6 for subsequent incident delirium and their associations with this outcome in Firth-penalized logistic regression models. These secondary analyses were intended for hypothesis generation and not for the development or validation of a clinical diagnostic test or prediction model.

## 2. Methods

### 2.1. Study Design and Population

**Figure 1:**
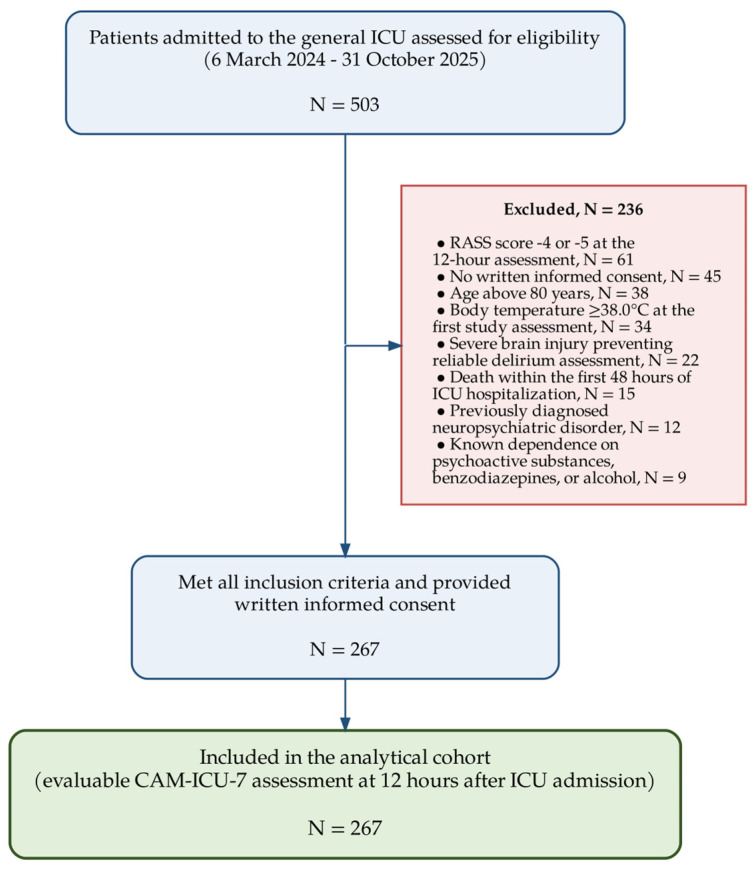
Flow diagram of patient screening, eligibility, and inclusion in the analytical cohort.

The study was a prospective observational cohort study conducted in a general intensive care unit. Consecutive adult patients admitted to the ICU with critical illness were screened for eligibility. Patients were recruited between 6 March 2024 and 31 October 2025. During this period, 503 patients admitted to the ICU were assessed for eligibility. The flow of patients from initial eligibility screening to inclusion in the analytical cohort is shown in Figure 1. Eligibility was not based on the ability to communicate verbally. Patients receiving invasive mechanical ventilation or sedation could be included provided that their level of arousal permitted a valid CAM-ICU-7 assessment.

Before each scheduled delirium assessment, the level of arousal was evaluated using the Richmond Agitation Sedation Scale. CAM-ICU-7 was administered when the RASS score was minus 3 or higher. Patients with RASS scores of −4 or −5 were considered unassessable at that assessment. Decision-making capacity for informed consent was evaluated separately from the level of arousal required for CAM-ICU-7 assessment.

Inclusion criteria were as follows:

- Age from 18 to 80 years;
- Admission to the general ICU because of critical illness;
- An evaluable CAM-ICU-7 assessment at 12 h after ICU admission, defined as a RASS score of minus 3 or higher;
- Written informed consent provided by the patient with decision-making capacity or by a legally authorized representative when the patient was temporarily unable to provide consent;
- No previously diagnosed neuropsychiatric disorder that could prevent reliable assessment of mental status;
- No known dependence on psychoactive substances, benzodiazepines, or alcohol;
- Body temperature below 38.0 degrees Celsius at the time of the first study assessment.

Exclusion criteria were as follows:

- Absence of written informed consent from the patient or, where applicable, a legally authorized representative;
- Age above 80 years;
- Severe brain injury preventing reliable delirium assessment;
- A RASS score of minus 4 or minus 5 at the 12 h assessment;
- Body temperature of at least 38.0 degrees Celsius at the time of the first study assessment;
- Death within the first 48 h of ICU hospitalization.

The fever exclusion criterion was specified in the original protocol to reduce domination of the biomarker signal by overt febrile illness at the time of the first study assessment. This criterion narrowed the clinical spectrum represented in the cohort and was considered when interpreting the generalizability of the findings.

Written informed consent was obtained directly from patients who had decision-making capacity. When a patient was temporarily unable to provide informed consent because of critical illness, impaired consciousness, delirium, mechanical ventilation, or sedation, written consent was obtained from a legally authorized representative in accordance with the protocol approved by the Ethics Committee. When decision-making capacity was subsequently recovered, the patient was asked to provide deferred written consent for continued participation and use of the collected data.

### 2.2. Study Procedure

The aim of this study was to prospectively evaluate the association between selected systemic inflammatory markers (C-reactive protein, interleukin-6, and procalcitonin) and the occurrence and severity of delirium in critically ill patients treated in the intensive care unit.

Clinical data and ICU-based assessments were collected prospectively during the ICU stay. Laboratory follow-up extended from ICU admission through the predefined 14 day assessment. Demographic characteristics, reasons for ICU admission, ICU length of stay, clinical outcomes, and selected inflammatory biomarkers were recorded. Patients were enrolled after eligibility screening and completion of the applicable written informed consent procedure. Patients with decision-making capacity provided consent personally. For patients temporarily unable to provide consent, written consent was obtained from a legally authorized representative, and deferred written consent was subsequently sought from the patient after recovery of decision-making capacity whenever possible. Before written informed consent was obtained, the patient or legally authorized representative received both oral and written information about the study, including its observational nature, study procedures, use of routinely collected clinical data, planned follow-up PCT sampling after ICU or hospital discharge when required, and the right to withdraw. They were given an opportunity to ask questions before signing the consent form. Mechanical ventilation, inability to speak, or the administration of sedation did not by themselves exclude participation.

The occurrence and severity of delirium were assessed using the Confusion Assessment Method for the Intensive Care Unit-7 at prespecified time points defined relative to ICU admission: 12 h and days 2, 4, 7, and 14. CAM-ICU-7 assessments were performed only while the patient remained in the ICU and had a RASS score of at least −3. No CAM-ICU-7 assessments were performed after ICU discharge.

Laboratory follow-up was conducted separately from delirium assessment. PCT was assessed at all five prespecified time points. When a patient had been discharged from the ICU before a scheduled PCT assessment, blood was collected either on another hospital ward, if the patient remained hospitalized, or during a scheduled outpatient laboratory visit. No telephone-based delirium assessments were performed.

This allowed for the assessment of the dynamics of changes in consciousness over time and the analysis of the association between inflammatory biomarker profiles and the occurrence of delirium. For grading delirium severity, the CAM-ICU-7 numerical scale was used. This scale converts responses from the CAM-ICU domains into a single numerical score ranging from 0 to 7, where: 0–2 = no delirium, 3–5 = mild to moderate delirium, 6–7 = severe delirium [14]. Delirium at any evaluable time point was defined as a CAM-ICU-7 score of 3 or greater. A case was defined as incident delirium if it was first identified after the 12 h assessment in a patient without baseline delirium. Immediately before each scheduled CAM-ICU-7 assessment, the level of arousal was evaluated using the Richmond Agitation Sedation Scale. CAM-ICU-7 was administered only when the RASS score was at least −3 or higher. Patients with RASS scores of −4 or −5 were classified as unable to be assessed at that time point, and no CAM-ICU-7 score was assigned. Mechanically ventilated patients remained eligible for assessment when the required level of arousal was present. For statistical analysis, delirium severity was categorized into three categories: no delirium, mild/moderate delirium, and severe delirium. Additionally, for binary analysis, patients were divided into a delirium group and a non-delirium group. The presence of delirium at any evaluable assessment during the in-ICU observation period, up to day 14, was considered an endpoint.

### 2.3. Laboratory Tests

CRP and IL-6 measurements were obtained as part of routine clinical care and were extracted from the hospital information system at the prespecified assessment times. CRP was reported in mg/L and measured by immunoturbidimetry. IL-6 was reported in pg/mL and measured by an enzyme-linked immunosorbent assay.

PCT was reported in ng/mL and measured by electrochemiluminescence using a Roche Elecsys analyzer. PCT measurements obtained during the ICU stay or during continued hospitalization on another hospital ward were extracted from the hospital information system. When a patient had been discharged from the hospital before a scheduled later PCT assessment, a follow-up blood sample was collected during a planned outpatient laboratory visit according to the study protocol. All PCT assessment times were defined relative to ICU admission, irrespective of the patient’s location at the time of blood sampling.

The analytic dataset contained repeated minimum reported values of 4 mg/L for CRP and 1.7 pg/mL for IL-6. These values were retained exactly as recorded. No substitution below an assay limit, transformation, or imputation was performed. Manufacturer-specific lower limits of detection or quantification were not available in the analytic export, and the observed minimum reported values are therefore described as reporting floors rather than analytical detection limits.

PCT was represented as an ordinal variable with three categories: below 0.10 ng/mL, 0.10 to 0.50 ng/mL, and above 0.50 ng/mL. PCT category values were available for all 267 patients at each of the five prespecified assessment times: 12 h and days 2, 4, 7, and 14.

The boundaries of these categories correspond to conventional clinical thresholds used in the hospital where the study was conducted to assess the likelihood of bacterial infection, where concentrations below 0.10 ng/mL are considered low, concentrations between 0.10 and 0.50 ng/mL are considered indeterminate, and concentrations above 0.50 ng/mL are considered predisposing to bacterial infection or sepsis.

For patients no longer treated in the ICU at a scheduled late assessment, the PCT measurement was obtained either during continued hospitalization on another ward or during a scheduled outpatient laboratory visit. All recorded PCT categories represented actual laboratory measurements. No imputation, replacement, extrapolation, or last-observation-carried-forward procedure was used.

### 2.4. Endpoints

The primary outcome was concurrent delirium severity measured by CAM-ICU-7 at each assessment time. Descriptive outcomes included the proportion of patients with no delirium, mild or moderate delirium, and severe delirium at each time point; the number of patients with delirium at any assessment; and the timing of the first delirium classification.

Secondary exploratory outcomes included concurrent and cross-time associations of CRP and IL-6 concentrations and ordinal PCT categories with CAM-ICU-7 severity at all five assessment times, as well as associations between inflammatory biomarkers and ICU length of stay. Cross-time correlations were interpreted as exploratory associations rather than evidence of prospective prediction. CAM-ICU-7 outcomes were available only during ICU treatment, whereas scheduled PCT follow-up could continue after ICU discharge. Consequently, later PCT measurements obtained outside the ICU were included in descriptive and serial PCT analyses but could contribute to concurrent biomarker-delirium analyses only when a corresponding evaluable CAM-ICU-7 assessment was available.

An additional secondary exploratory outcome was the apparent within-sample discrimination of baseline CRP and IL-6 for subsequent incident delirium among patients without delirium at the 12 h assessment. The ROC analysis, exploratory cutoff selection, and Firth-penalized regression models were not intended to establish or validate a clinical diagnostic test or prediction model.

### 2.5. Statistical Analysis

Descriptive analyses and Spearman rank correlations were performed using Statistica 13.3 PL software, TIBCO Software Inc., Palo Alto, CA, USA. Receiver operating characteristic curve analyses, bootstrapping procedures, and logistic regression analyses with Firth penalty were performed using Python 3.14.

The distributions of quantitative variables were assessed using the Shapiro–Wilk test. Variables with approximately normal distributions were summarized using the mean and standard deviation. Variables with asymmetric distributions were summarized using the median and the minimum-to-maximum range. Categorical variables were presented as numbers and percentages.

Missing observations were not imputed. Analyses were conducted using available observations, and the number of patients included was reported separately for each assessment time and analysis. Percentages describing delirium severity were calculated using the number of available assessments at the corresponding time point as the denominator.

Delirium severity was analyzed as an ordinal variable with three categories: no delirium, mild or moderate delirium, and severe delirium. CRP and IL-6 were analyzed as continuous variables. PCT was analyzed as a three-level ordinal variable corresponding to concentrations below 0.10 ng/mL, concentrations from 0.10 to 0.50 ng/mL, and concentrations above 0.50 ng/mL. PCT categories from all five assessment times were included in the analyses.

Spearman rank correlation coefficients were used to evaluate serial associations between measurements of each inflammatory marker and associations between each biomarker and delirium severity across all five assessment times. For PCT, the analyses were based on the three-level ordinal category rather than on exact continuous concentrations. Concurrent and cross-time PCT correlations were calculated for measurements obtained at 12 h and on days 2, 4, 7, and 14.

Associations between inflammatory biomarkers and ICU length of stay were evaluated using Spearman rank correlation coefficients and were considered exploratory. PCT analyses included 267 patients at each assessment time. For correlations with delirium severity, only paired CAM-ICU-7 and biomarker observations available at the corresponding assessment time were used. PCT measurements obtained after ICU discharge without a corresponding CAM-ICU-7 assessment were retained for descriptive and serial PCT analyses but did not contribute to concurrent correlations with delirium severity. The sample size for each concurrent correlation was determined by the number of patients with both an evaluable CAM-ICU-7 assessment and a corresponding biomarker measurement at that time point.

The prospective ROC analysis was restricted to patients without delirium at the assessment performed 12 h after ICU admission. Patients with delirium at this baseline assessment were excluded. Incident delirium was defined as the first subsequent recording of mild, moderate, or severe delirium on day 2, day 4, day 7, or day 14.

Baseline CRP and IL-6 concentrations were evaluated for their ability to discriminate between patients who subsequently developed incident delirium and those who did not. The prospective ROC analysis included 254 patients, of whom 25 developed incident delirium and 229 remained free of incident delirium.

Areas under the receiver operating characteristic curves were calculated nonparametrically. Their 95% confidence intervals were estimated using 10,000 stratified bootstrap samples while preserving the proportions of patients with and without incident delirium. Exploratory cutoff values were selected by maximizing the Youden index. Because the cutoff values were selected and evaluated in the same cohort, the resulting sensitivity and specificity estimates represent apparent within-sample performance. They were not obtained from a separate validation sample. Sensitivity and specificity were calculated at the selected cutoff values, with corresponding exact binomial 95% confidence intervals. The difference between the CRP and IL-6 areas under the curve was evaluated using paired bootstrap resampling because both biomarkers were measured in the same patients. Bootstrap resampling was used to estimate confidence intervals and compare the AUC values. It was not used to produce optimism-corrected estimates of predictive performance. Model calibration, decision curve-based net clinical benefit, and clinical utility were not evaluated.

Because only 25 incident delirium events occurred and baseline CRP concentrations showed strong separation between patients who did and did not subsequently develop delirium, associations with incident delirium were estimated using Firth-penalized logistic regression.

Separate models were fitted for CRP and IL-6, with each model adjusted for age and sex. A joint model included CRP, IL-6, age, and sex simultaneously. Odds ratios for CRP were expressed per 5 mg/L increase, odds ratios for IL-6 per 5 pg/mL increase, and odds ratios for age per 10-year increase. Female sex was used as the reference category. Results were reported as adjusted odds ratios with 95% confidence intervals derived from the profile-penalized likelihood. Two-sided *p*-values were calculated using penalized likelihood ratio tests comparing the fitted model with the model in which the corresponding regression coefficient is constrained to zero. Because profile-penalized likelihood intervals may be asymmetric, *p*-values were not derived from Wald standard errors and cannot be reliably reconstructed from rounded confidence limits.

Although PCT was included in descriptive and Spearman rank correlation analyses at all five assessment times, it was not included in the prospective ROC or Firth-penalized logistic regression analyses because only ordinal categories, rather than complete continuous PCT measurements, were available in the analytic dataset.

All statistical tests were two-sided. A *p*-value below 0.05 was considered statistically significant. Correlation analyses across multiple assessment times were considered exploratory, with emphasis on the magnitude and direction of the correlation coefficients.

### 2.6. Ethical Considerations

The study was conducted in accordance with the principles of the Declaration of Helsinki and applicable national regulations. Approval was obtained before initiation of the study from the Bioethics Committee of the District Medical Chamber in Gdańsk, approval number KB 6A/24, dated 5 March 2024.

Written informed consent was obtained directly from all participants who had decision-making capacity. When a patient was temporarily unable to provide informed consent because of critical illness, impaired consciousness, delirium, mechanical ventilation, or sedation, written consent was obtained from a legally authorized representative in accordance with the approved study protocol. When the patient subsequently recovered decision-making capacity, deferred written consent was sought whenever possible. The approved study protocol and informed consent procedure included planned follow-up PCT sampling after ICU discharge, as needed to complete the predefined laboratory assessment schedule. Patients who had been discharged from the hospital before a scheduled later PCT assessment could return for an outpatient blood draw. No CAM-ICU-7 assessment was performed during these outpatient visits.

The ability to provide informed consent was assessed separately from eligibility for delirium assessment. Mechanical ventilation or inability to communicate verbally did not automatically exclude participation. Eligibility for each CAM-ICU-7 assessment required a RASS score of −3 or higher. No CAM-ICU-7 assessment was performed when the patient had a RASS score of −4 or −5.

## 3. Results

### 3.1. Demographic Analysis of the Study Group

The study included 267 patients admitted to the ICU. Men accounted for 58.80% of the cohort (157 of 267 patients), and women accounted for 41.20% (110 of 267 patients). The largest age group was 61–70 years (90 of 267 patients, 33.71%), followed by 41–50 years (68 of 267 patients, 25.47%), 51–60 years (55 of 267 patients, 20.60%), 30 years or younger (25 of 267 patients, 9.36%), 71 years or older (15 of 267 patients, 5.62%), and 31–40 years (14 of 267 patients, 5.24%). The most common reason for ICU admission was circulatory failure (93 of 267 patients, 34.83%). Respiratory failure and acute renal failure each accounted for 43 cases (16.10%), gastrointestinal bleeding for 34 cases (12.73%), multi-organ trauma for 24 cases (8.99%), hypothermia for 19 cases (7.12%), and acute pancreatitis for 11 cases (4.12%). The association between reason for ICU admission and delirium status is presented, for exploratory purposes, in Supplementary Table S9. Overall, 255 patients (95.51%) were discharged from the ICU, whereas 12 patients (4.49%) died. ICU length of stay ranged from 3 to 45 days (Table 1).

### 3.2. Analysis of the Incidence of Delirium in the ICU Patient Group

All 267 patients included in the analytical cohort had an evaluable CAM-ICU-7 assessment at 12 h after ICU admission. The lower denominators at the one-week and two-week assessments represent the number of available and evaluable assessments at those time points. Delirium severity was assessed using CAM-ICU-7 at the prespecified in-ICU assessment times. Mild or moderate delirium was most frequent on day 2, affecting 29 of 267 patients (10.9%). Severe delirium was most frequent on day 4, affecting 16 of 267 patients (6.0%). Thirteen patients had delirium at the 12 h assessment, and 25 additional patients first met the delirium criterion on day 2. No new incident cases were identified after day 2.

Because all 25 incident cases were first identified on day 2, the prospective ROC and regression analyses described in Section 3.5 and Section 3.6 in practice discriminate delirium onset within an approximately 36 h horizon, from the 12 h biomarker measurement to the day-2 assessment, rather than across the full ICU stay.

Detailed results of the analysis are presented in Table 2.

The correlation between delirium severity scores across available in-ICU assessment times was evaluated using Spearman’s rank correlation coefficient. The analysis revealed positive and statistically significant correlations among delirium severity scores obtained at the available in-ICU assessment times, indicating temporal consistency of delirium severity within the observation period. Detailed results are presented in Table 3.

### 3.3. Analysis of Inflammatory Parameters in the Study Cohort

Selected inflammatory biomarkers, including CRP, PCT, and IL-6, were analyzed at the prespecified assessment times. CRP and IL-6 measurements were obtained as part of routine clinical care and extracted from the hospital information system. PCT results were obtained either during the ICU stay, during continued hospitalization on another ward, or during a planned outpatient laboratory visit when the patient had already been discharged from the hospital. All biomarker data were used in accordance with the informed consent procedure described in Section 2.1, Section 2.2 and Section 2.6. The highest proportion of patients with CRP concentrations above 5 mg/L was observed at 12 h after ICU admission and on day 2, amounting to 16.10% at both assessment times (43 of 267 patients). The lowest proportion was observed at 1 week, when 12.55% of patients had CRP concentrations above 5 mg/L (31 of 247 patients). At 2 weeks, the corresponding proportion was 13.40% (26 of 194 patients). Detailed results are presented in Table S1 in the Supplementary Materials.

The analysis also examined the significance of correlations among individual CRP levels using Spearman’s R. CRP levels were strongly and statistically significantly positively correlated across the different days of hospitalization (Table S2—Supplementary Materials).

Procalcitonin was recorded and analyzed as an ordinal variable with three categories: below 0.10 ng/mL, from 0.10 to 0.50 ng/mL, and above 0.50 ng/mL. At the 12 h assessment, 264 patients were classified in the lowest category, 3 in the intermediate category, and none in the highest category. On day 2, the corresponding numbers were 246, 3, and 18 patients. On day 4, 245 patients were classified in the lowest category and 22 in the highest category, with no patients in the intermediate category. At 1 week, 244 patients were classified in the lowest category, 1 in the intermediate category, and 22 in the highest category. At 2 weeks, the corresponding numbers were 248, 1, and 18 patients. PCT category data were available for all 267 patients at each assessment time. For patients discharged from the ICU before a scheduled later PCT assessment, the blood sample was obtained either on another hospital ward, when hospitalization continued, or during a planned outpatient laboratory visit after hospital discharge. No patient died before completion of the day 14 PCT assessment. All reported PCT categories were actual laboratory measurements obtained at the corresponding assessment time, and no imputation or carry-forward procedures were used. Detailed distributions are presented in Table S3 in the Supplementary Materials.

Correlations between ordinal PCT categories across assessment times were evaluated using Spearman rank correlation coefficients. All serial correlations were positive and ranged from 0.349 to 0.976. The strongest correlations were observed among PCT categories recorded from day 2 onward. Detailed results are presented in Table S4 in the Supplementary Materials.

Interleukin-6 concentrations were also analyzed at subsequent time points during hospitalization among patients treated in the Intensive Care Unit. Interleukin-6 is a key mediator of the inflammatory response, playing a significant role in the initiation and maintenance of the acute phase reaction. For descriptive purposes, IL-6 concentrations were categorized as 10 pg/mL or lower and greater than 10 pg/mL. Values greater than 10 pg/mL were classified as elevated. In the analyzed cohort, the highest proportion of patients with elevated IL-6 concentrations was observed at 1 week, amounting to 11.16% (27 of 242 patients). At the 12 h assessment, elevated IL-6 concentrations were observed in 4.12% of patients (11 of 267 patients). Detailed results are presented in Table S5 in the Supplementary Materials.

### 3.4. Correlation Analysis Between Selected Inflammatory Markers and Delirium

Spearman rank correlation analysis was used to explore concurrent and cross-time associations between inflammatory biomarkers and delirium severity across the five predefined assessment times. CRP and IL-6 were analyzed as continuous variables, whereas PCT was analyzed as a three-level ordinal variable.

Positive correlations were observed between all three inflammatory biomarkers and CAM-ICU-7 severity. Concurrent correlations between ordinal PCT categories and CAM-ICU-7 severity were 0.304 at 12 h, 0.666 on day 2, 0.711 on day 4, 0.720 at 1 week, and 0.329 at 2 weeks. The magnitude and relative ordering of the associations varied by biomarker and assessment time.

Because the analysis included both concurrent and cross-time correlations, the coefficients should be interpreted as exploratory rank associations within the observed dataset and not as evidence that a biomarker measurement prospectively predicted delirium at a later assessment. Detailed results are presented in Table 4.

The associations between inflammatory biomarkers measured 12 h after ICU admission and CAM-ICU-7 severity at subsequent assessment times varied by biomarker and assessment time. Baseline CRP showed the largest cross-time coefficients for CAM-ICU-7 severity, assessed on days 2, 4, and 7. These cross-time associations were exploratory and should not be interpreted as evidence of prospective prediction.

The relative strength of the concurrent biomarker associations also varied across assessment times. IL-6 had the largest concurrent coefficient at 12 h. CRP had the largest concurrent coefficients on days 2 and 4. At 1 week, the concurrent coefficients for CRP, IL-6, and PCT were nearly identical, whereas at 2 weeks, PCT had the largest coefficient. PCT showed moderate-to-strong concurrent correlations with CAM-ICU-7 severity on days 2, 4, and 7, with weaker correlations at 12 h and 2 weeks. Overall, the results do not support a fixed hierarchy among CRP, IL-6, and PCT across the observation period.

Associations between inflammatory biomarkers and ICU length of stay were explored using Spearman rank correlation coefficients. CRP was not significantly associated with ICU length of stay at 12 h, on day 2, or on day 4. Weak positive correlations were observed at 1 week rho = 0.128, *p* = 0.045 and at 2 weeks rho = 0.185, *p* = 0.010. IL-6 was not significantly associated with ICU length of stay at 12 h, day 2, or day 4. Weak positive correlations were observed at 1 week, rho = 0.148, *p* = 0.021, and at 2 weeks, rho = 0.186, *p* = 0.010.

Ordinal PCT categories were positively correlated with ICU length of stay at all five assessment times. The Spearman coefficients were 0.151 at 12 h, 0.214 on day 2, 0.219 on day 4, 0.237 at 1 week, and 0.281 at 2 weeks. The corresponding *p* values were 0.013 at 12 h and below 0.001 at each subsequent assessment. All PCT analyses included 267 patients.

The magnitude of the PCT correlations was weak. These findings demonstrate monotonic associations between higher PCT categories and longer ICU stay, but they do not establish temporal direction, causality, or an independent prognostic effect because no multivariable model of ICU length of stay was performed. Details are presented in Table 5.

### 3.5. Secondary Exploratory ROC Analysis

The secondary exploratory ROC analysis included 254 patients without delirium at the 12 h assessment. Incident delirium subsequently occurred in 25 patients, whereas 229 patients remained free of incident delirium.

Baseline CRP showed high apparent within-sample discrimination for subsequent incident delirium, with an AUC of 0.969 and a bootstrap 95% confidence interval from 0.923 to 0.997. An exploratory cutoff of at least 8.0 mg/L, selected by maximizing the Youden index in the same cohort, identified 24 of the 25 incident cases, corresponding to an apparent sensitivity of 96.0%. It correctly classified 223 of the 229 patients without incident delirium, corresponding to an apparent specificity of 97.4%.

This apparent discrimination should be interpreted in light of the underlying biomarker and outcome distributions (Supplementary Tables S1, S3 and S5): CRP concentrations that were equal to or below the floor level of 5 mg/L in approximately 84% of patients, IL-6 concentrations were equal to or below 10 pg/mL in approximately 90% of patients, and PCT was in the lowest ordinal category in approximately 92% of patients, whereas delirium was absent in approximately 86% of assessments. Because both biomarker exposure and the delirium outcome were dominated by concordant floor values, the reported AUC and cutoff point of 8.0 mg/L largely reflect a near-binary separation between floor and elevated CRP concentrations rather than evidence of a graded biomarker-severity association. In essence, this finding is that the same 14% to 16% of patients had both elevated inflammatory markers and delirium; this is a reasonable but modest finding, and the highly discriminatory language used elsewhere in this manuscript should be read with this caveat in mind.

Baseline IL-6 showed lower apparent within-sample discrimination, with an AUC of 0.653 and a bootstrap 95% confidence interval ranging from 0.569 to 0.750. The internally selected exploratory cutoff of at least 2.1 pg/mL had an apparent sensitivity of 32.0% and an apparent specificity of 98.7%. The absolute difference between the CRP and IL-6 AUC values was 0.316, with a paired bootstrap 95% confidence interval from 0.219 to 0.403.

All AUC, cutoff, sensitivity, and specificity estimates were derived and evaluated within a single-center cohort. They therefore represent apparent performance and may be affected by optimism, particularly given the limited number of 25 incident delirium events. No separate validation cohort was available. Calibration, net clinical benefit, and clinical utility were not assessed. The reported cutoff values should therefore be regarded as hypothesis-generating rather than clinically validated thresholds.

Detailed results are presented in Supplementary Table S7.

### 3.6. Firth-Penalized Logistic Regression

In the separate Firth-penalized logistic regression model adjusted for age and sex, baseline CRP was associated with subsequent incident delirium. Each 5 mg/L increase in CRP was associated with an adjusted odds ratio of 10.30, with a penalized 95% confidence interval from 4.66 to 29.04 and a *p*-value below 0.001. Neither age nor sex was significantly associated with incident delirium in this model.

Baseline IL-6 was also associated with incident delirium in the separate model adjusted for age and sex. Each 5 pg/mL increase in baseline IL-6 was associated with an adjusted odds ratio of 2.79, with a profile-penalized likelihood 95% confidence interval of 1.28 to 7.52. The penalized likelihood ratio test yielded χ^2^ = 12.81 with one degree of freedom and a two-sided *p*-value of 0.00034. Age and sex were not significantly associated with the outcome.

In the joint model including biomarkers, age, and sex, CRP remained associated with subsequent incident delirium after adjustment for IL-6, age, and sex. Each 5 mg/L increase in baseline CRP was associated with an adjusted odds ratio of 10.75, with a 95% confidence interval from 4.50 to 33.18 and a *p*-value below 0.001. In contrast, IL-6 was no longer independently associated with incident delirium after adjustment for CRP. The adjusted odds ratio per 5 pg/mL increase in IL-6 was 0.81 (95% CI, 0.42 to 2.04; *p* = 0.510). Neither age nor sex was statistically significant in the joint model.

The joint model included 25 incident events across four covariates, corresponding to approximately six events per variable, which limits the precision and stability of all estimates in this model, including the IL-6 coefficient described above.

The attenuation of the IL-6 association after inclusion of CRP indicates that IL-6 did not provide independent information beyond CRP in this dataset. The large CRP odds ratios reflect the strong separation in baseline CRP concentrations between patients who did and those who did not subsequently develop delirium. They should not be interpreted as evidence of causality or as externally validated estimates of clinical risk. These regression results describe associations within the analyzed cohort and should not be interpreted as validated estimates of individual clinical risk. The limited number of incident events, restricted covariate adjustment, and absence of external validation constrain their prognostic interpretation.

Detailed results of the Firth-penalized logistic regression models are presented in Supplementary Table S8.

## 4. Discussion

This prospective observational cohort study evaluated the relationships between systemic inflammatory biomarkers and delirium in critically ill adults using both serial exploratory analyses and a prospectively defined incident delirium endpoint. Delirium was identified in 38 of 267 patients. Thirteen patients had delirium at the 12 h assessment, while 25 additional patients subsequently developed incident delirium, with all incident cases first identified on day 2. The principal secondary exploratory finding was that CRP measured 12 h after ICU admission showed high apparent within-sample discrimination for subsequent incident delirium and remained associated with the outcome after adjustment for IL-6, age, and sex in the specified Firth model. In contrast, IL-6 demonstrated limited discriminative power and was no longer independently associated with incident delirium in the joint model. Ordinal PCT categories showed positive exploratory associations with concurrent delirium severity at all five assessment times and weak positive correlations with ICU length of stay. PCT was not evaluated in the prospective ROC or Firth regression analyses.

These findings are biologically plausible within current models of delirium pathophysiology. Severe systemic insults, including infection, tissue injury, hypoxia, and organ dysfunction, may initiate a peripheral inflammatory response that contributes to blood–brain barrier dysfunction, microglial activation, endothelial injury, altered cerebral metabolism, and neurotransmitter imbalance [15,16,17,18]. Nevertheless, circulating inflammatory biomarkers are nonspecific and are influenced by numerous factors other than acute brain dysfunction, including the underlying reason for ICU admission, the severity and source of infection, tissue damage, organ failure, therapeutic interventions, and preexisting comorbidity [16,17]. The associations observed in this study should therefore not be interpreted as evidence that elevations in CRP, IL-6, or PCT directly caused delirium.

The temporal distribution of delirium in the present cohort is also relevant to interpreting the biomarker findings. Mild or moderate delirium was most frequent on day 2, while the highest proportion of severe delirium was observed on day 4. All incident cases were first identified on day 2, indicating that subsequent assessments primarily reflected persistence, resolution, or changes in severity rather than the occurrence of new delirium. This early temporal pattern is compatible with models in which the neurological consequences of systemic inflammation become clinically apparent within the first 24 to 72 h after a major physiological insult [15,19]. Alternatively, the complete absence of new-onset delirium after day 2, despite a median ICU stay of 21 days, may partly reflect the eligibility criteria rather than a general temporal pattern of delirium in critical illness: patients with RASS scores of −4 or −5, fever at the first assessment, age above 80 years, and death within the first 48 h were excluded from the analytical cohort (see Limitations, fourth point), which may have removed much of the population at risk of later-onset delirium. However, the serial Spearman analyses included both concurrent and cross-time associations. Their magnitude varied with the timing of both the biomarker measurement and the delirium assessment. These correlations should therefore be considered exploratory and should not be interpreted as demonstrating a uniform hierarchy among the three biomarkers or as establishing prospective prediction.

CRP provided the clearest prospective signal in the present study. This result is consistent with previous reports linking elevated CRP concentrations with delirium in surgical and critically ill populations. Knaak et al. found that higher preoperative CRP concentrations were independently associated with postoperative delirium and postoperative neurocognitive disorders [20]. Ren et al. reported an association between elevated CRP and both the occurrence and severity of delirium following cervical or lumbar surgery [21]. Qin et al. also observed a positive association between high-sensitivity CRP concentrations and postoperative delirium after general anesthesia [22]. In addition, a recent meta-analysis reported that elevated CRP was associated with a higher risk of delirium across a broad range of clinical populations, although considerable between-study heterogeneity was present [23]. The pooled effect size in that meta-analysis, however, was considerably smaller than the association observed in the present study, and no significant association remained after adjustment in the critically ill, septic, or respiratory-failure subgroups. The much larger effect size and discrimination observed in our cohort therefore most plausibly reflect the floor-dominated, single-center distribution described in Section 3.5 rather than a stronger true biological effect and should not be interpreted as corroborated by this meta-analysis. Our previous retrospective analysis similarly demonstrated an association between CRP and postoperative delirium following cardiac and neurosurgical procedures [24].

Direct comparison of effect sizes and thresholds across these studies is difficult because of differences in patient populations, clinical settings, timing of blood sampling, biomarker assays, delirium definitions, and covariate adjustment. The exploratory CRP cutoff identified in the present study should therefore not be interpreted as equivalent to thresholds reported in perioperative studies or as a universally applicable clinical threshold. Rather, the consistency of the overall direction of association supports further investigation of CRP as one component of a broader delirium risk assessment strategy.

In the secondary exploratory ROC analysis, baseline CRP achieved an AUC of 0.969, with a 95% bootstrap confidence interval of 0.923 to 0.997. The internally selected exploratory cutoff of at least 8.0 mg/L had an apparent sensitivity of 96.0% and an apparent specificity of 97.4% within the analyzed cohort. These values indicate high apparent within-sample discrimination, but they should not be interpreted as validated diagnostic performance. As noted in Section 3.5, this apparent discrimination is best understood as a near-binary separation between floored and elevated CRP values, driven by the concordant floor distribution of both the biomarkers and the delirium outcome, rather than as a graded diagnostic gradient. The cutoff was selected and evaluated in the same cohort, which creates a risk of optimistic performance estimates. Bootstrap resampling was used to estimate confidence intervals rather than to provide an optimism-corrected validation of the cutoff. Furthermore, only 25 incident delirium events occurred, model calibration was not assessed, and neither decision curve-based net clinical benefit nor clinical utility was evaluated. The cutoff and performance estimates therefore require validation in independent external cohorts before they can be considered for clinical use.

Firth-penalized logistic regression was selected to reduce the small-sample and separation-related biases that could affect conventional maximum-likelihood estimates. In the joint model, each 5 mg/L increase in baseline CRP was associated with an adjusted odds ratio of 10.75. The corresponding confidence interval was wide, ranging from 4.50 to 33.18, reflecting uncertainty due to the limited number of incident events and the strong separation of CRP values between outcome groups. This estimate should not be interpreted as a causal effect, as an absolute increase in individual risk, or as an externally validated measure of clinical prognosis. It describes the association observed in the present cohort using the specified CRP scale.

The findings for IL-6 were less consistent. In the separate model adjusted for age and sex, each 5 pg/mL increase in IL-6 was associated with higher odds of incident delirium. However, IL-6 demonstrated limited discrimination in ROC analysis, with an AUC of 0.653, and the exploratory cutoff had a sensitivity of only 32.0%. After CRP and IL-6 were entered simultaneously into the Firth model, IL-6 was no longer independently associated with incident delirium. This attenuation suggests that the information represented by IL-6 overlapped with that conveyed by CRP, or that its association was less stable after mutual adjustment.

With only 25 events across four covariates in the joint model (approximately six events per variable) and strong co-elevation of CRP and IL-6, this sign reversal—from a significant positive association in the separate model to a non-significant point estimate below unity in the joint model—is characteristic of collinearity-driven instability rather than clean attenuation. The joint-model IL-6 estimate should therefore not be interpreted as evidence that IL-6 conveys no independent information; it is presented for completeness alongside the more stable separate-model estimate.

Previous studies have also reported heterogeneous findings for IL-6. Lv et al. identified plasma IL-6 as a potential biomarker of postoperative delirium following surgery for acute type A aortic dissection [25]. In contrast, observational research on low-grade systemic inflammation and perioperative neurocognitive outcomes has demonstrated that associations may differ substantially among inflammatory markers and patient populations [26]. IL-6 has a more rapid and dynamic response than CRP, and its measured concentration may depend strongly on the interval between the initiating insult, blood collection, and delirium assessment. Moreover, related research from the BioCog program indicates that perioperative neurocognitive outcomes may involve network-level alterations and mechanisms that extend beyond circulating inflammation alone [27]. These considerations may explain why IL-6 was associated with incident delirium in the separate model but did not provide independent information once CRP was included.

The PCT results require a distinct interpretation. PCT was represented as a three-level ordinal variable and was evaluated at all five prespecified assessment times. Higher ordinal PCT categories were positively associated with concurrent CAM-ICU-7 severity at each time point. The correlations were weakest at 12 h and 2 weeks and strongest on days 4 and 7.

This distribution should be interpreted with caution: more than 90% of patients remained in the lowest PCT category at every assessment, and the intermediate category (0.10–0.50 ng/mL) was nearly empty (Supplementary Table S3). In a mixed general ICU population that included patients admitted for acute renal failure, a condition that can elevate PCT independently of infection, this near-absent intermediate band and the sustained predominance of the lowest category over two weeks warrant caution when interpreting the ordinal PCT associations reported above.

PCT categories also showed weak positive correlations with ICU length of stay at all five assessment times. These findings are consistent with PCT reflecting a systemic host response to bacterial infection and sepsis. Experimental evidence has demonstrated widespread calcitonin precursor expression across multiple tissues during sepsis, supporting the interpretation of circulating PCT as a systemic inflammatory biomarker [28,29]. However, because PCT was not included in the prospective ROC analysis or the Firth regression models, the present study does not demonstrate that PCT independently discriminates or predicts incident delirium.

Song et al. reported that postoperative PCT, CRP, and IL-6 concentrations were associated with delirium in ICU patients, and that PCT remained independently significant in their multivariable analysis [13]. Their findings cannot be directly compared with the present study because of differences in the clinical population, biomarker timing, outcome definition, data representation, and statistical modeling. In particular, the use of ordinal PCT categories in the current dataset resulted in loss of quantitative information and prevented an equivalent continuous prospective analysis. The observed PCT correlations should therefore be regarded as hypothesis-generating.

The correlations between ordinal PCT categories and ICU length of stay were weak but consistently positive. The Spearman coefficients increased from 0.151 at 12 h to 0.281 at 2 weeks, and each analysis included all 267 patients. These findings indicate that patients in higher PCT categories tended to have longer ICU stays. However, the analysis was based on unadjusted rank correlations. It does not determine the direction of the relationship or establish that PCT independently predicts prolonged ICU hospitalization. Confirmation would require an appropriately adjusted regression model with ICU length of stay as the outcome.

Several features strengthen the present study. Clinical and biomarker data were collected at predefined assessment times, and delirium was assessed serially using CAM ICU. The analysis distinguished between delirium already present at the 12 h assessment and delirium first identified after the 12 h assessment. This distinction made it possible to conduct a genuinely prospective ROC and regression analysis using baseline biomarker concentrations. Bootstrap confidence intervals were used for the ROC estimates, while Firth-penalized logistic regression was used to reduce instability associated with the small number of events and strong biomarker separation. The separate presentation of exploratory serial correlations and prospective analyses of incident delirium also reduced the risk of interpreting concurrent associations as evidence of prediction.

The study nevertheless has important limitations. First, it was conducted in a single general ICU and included patients with heterogeneous reasons for admission. Although this broadens the cohort’s clinical breadth, it also introduces variability in the causes, magnitude, and duration of systemic inflammation. The associations may differ in more homogeneous populations, such as patients with sepsis, trauma, postoperative critical illness, or respiratory failure.

Second, only 25 incident delirium events were available for the secondary exploratory prospective analyses. Firth penalization reduces bias related to sparse events and separation but does not eliminate statistical uncertainty or establish model validity. The ROC cutoff, sensitivity, specificity, and regression estimates were derived and evaluated in the same cohort and therefore represent apparent within-sample performance. Bootstrap resampling was used to estimate confidence intervals but was not used to provide optimism-corrected performance estimates. No separate validation cohort was available. Model calibration, decision curve-based net clinical benefit, and clinical utility were not assessed. Consequently, the apparent magnitude of discrimination and association may be overestimated, and none of the reported thresholds or effect estimates should be applied directly in clinical practice.

Third, the adjusted models included age and sex but excluded several clinically relevant potential confounders. Disease severity scores, such as APACHE II or SOFA, mechanical ventilation, sedative and analgesic exposure, infection source, microbiological findings, organ dysfunction, sleep disruption, and other recognized delirium risk factors were not included. Residual confounding therefore remains likely, and the independent contribution of systemic inflammation cannot be separated fully from the severity of critical illness.

Fourth, inclusion in the analytical cohort required an evaluable CAM-ICU-7 assessment at 12 h after ICU admission. Patients with deep sedation or coma, defined as a RASS score of minus 4 or minus 5 at that assessment, were therefore not included. Mechanical ventilation itself was not an exclusion criterion as long as the patient remained sufficiently arousable for CAM-ICU-7 assessment. In addition, patients older than 80 years, patients with severe brain injury, patients with fever at the first assessment, and patients who died within the first 48 h were excluded. These criteria limit generalizability to the most severely ill ICU populations and may partly explain the relatively low mortality and delirium frequency observed in the cohort.

Fifth, PCT was analyzed as an ordinal variable rather than a continuous measure. This approach allowed the available data to be used consistently but reduced statistical information and prevented direct comparison with the continuous CRP and IL-6 analyses. In addition, the large number of exploratory correlations across biomarkers and assessment times increases the possibility of chance findings. The correlation matrix should therefore be interpreted primarily by the magnitude, direction, timing, and consistency of associations rather than by individual significance tests.

Sixth, the ascertainment of later biomarkers varied across the markers analyzed. The number of available CAM-ICU-7, CRP, and IL-6 assessments decreased at later time points. In contrast, scheduled ordinal PCT measurements were completed for all 267 patients, including patients who had already been discharged from the ICU. In such cases, the later PCT sample was obtained either on another hospital ward or during a scheduled outpatient laboratory visit. Consequently, descriptive and serial PCT analyses included measurements obtained in different clinical settings, whereas concurrent associations between PCT and CAM-ICU-7 severity were restricted to patients with paired assessments obtained during the ICU stay. Differences in the clinical setting and patient condition at the time of later PCT sampling may have introduced additional heterogeneity and should be considered when interpreting the longitudinal PCT findings. The exact proportion of later PCT measurements obtained outside the ICU (on another hospital ward or during a scheduled outpatient visit) was not systematically recorded in the analytic dataset and cannot be reported; this represents an additional limitation of the longitudinal PCT ascertainment that should be considered when interpreting Supplementary Table S3.

From a clinical perspective, the present findings support further investigation of early CRP measurement as a potential component of delirium risk enrichment. They do not support the use of CRP as a stand-alone diagnostic test, screening threshold, or clinical decision rule. The internally selected cutoff of at least 8.0 mg/L should not be applied to individual patients because it has not undergone external validation, calibration assessment, or evaluation of clinical benefit. Any future risk model should combine biomarker information with established clinical predictors, including age, baseline vulnerability, disease severity, organ dysfunction, mechanical ventilation, sedation exposure, and repeated clinical delirium screening, and should undergo full validation in independent multicenter cohorts before clinical implementation.

In conclusion, systemic inflammatory biomarkers were associated with delirium severity in this ICU cohort, but their clinical and statistical roles differed. In a secondary exploratory analysis, baseline CRP showed high apparent within-sample discrimination for subsequent incident delirium and remained associated with the outcome in the specified Firth model. IL-6 showed lower apparent discrimination and was no longer associated after adjustment for CRP. Ordinal PCT categories were associated with concurrent delirium severity and, weakly, with ICU length of stay, but were not evaluated as prospective predictors. None of these findings establishes a validated diagnostic test or clinical prediction model.

## 5. Conclusions

In this prospective single-center ICU cohort, CRP, IL-6, and ordinal PCT categories were positively associated with concurrent delirium severity, although the magnitude of these associations varied across assessment times.

In secondary exploratory analyses involving 254 patients and 25 incident delirium events, baseline CRP showed high apparent within-sample discrimination for subsequent incident delirium and remained associated with the outcome after adjustment for IL-6, age, and sex in the specified Firth model. IL-6 showed lower apparent discrimination and did not remain associated after CRP was included in the joint model. These analyses describe findings within the derivation cohort and do not establish validated diagnostic or prognostic performance.

Ordinal PCT categories were positively associated with concurrent delirium severity and showed weak correlations with ICU length of stay. PCT was not included in the prospective ROC or Firth regression analyses, and no adjusted model of ICU length of stay was performed.

The internally selected CRP cutoff, sensitivity, specificity, AUC, and regression effect estimates should be regarded as hypothesis-generating. Model calibration, optimism-corrected performance, net clinical benefit, and clinical utility were not evaluated. CRP should not be used as a stand-alone diagnostic test or clinical decision threshold based on these results. Independent external validation in adequately powered multicenter cohorts is required before any clinical implementation.

## Figures and Tables

**Table 1:** Characteristics of the analyzed indicators describing the studied group.

Indicator	Group	N	%	Mean ± SD (Min–Max); Median	
Age [years]	Total	267	100.00%	52.61 ± 13.94 (18–78) ME = 56	
	30 years and younger	25	9.36%		
	31–40 years	14	5.24%		
	41–50 years	68	25.47%		
	51–60 years	55	20.60%		
	61–70 years	90	33.71%		
	71 years and older	15	5.62%		
Sex	Female	110	41.20%		
	Male	157	58.80%		
ICU length of stay [days]	Total	267	100.00%	19.89 ± 9.32 (3–45) ME = 21	
	Hypothermia	19	7.12%	5.95 ± 2.70 (3–12) ME = 5	
	Gastrointestinal bleeding	34	12.73%	11.59 ± 6.42 (4–24) ME = 9	
	Circulatory failure	93	34.83%	25.27 ± 6.68 (9–45) ME = 25	
	Respiratory failure	43	16.10%	24.95 ± 6.98 (11–37) ME = 26	
	Acute renal failure	43	16.10%	13.65 ± 6.07 (6–31) ME = 12	
	Acute pancreatitis	11	4.12%	19.27 ± 11.47 (8–45) ME = 17	
	Multi-organ trauma	24	8.99%	24.29 ± 5.38 (17–35) ME = 22	
Death	No	255	95.51%		
	Yes	12	4.49%		

N—number of patients; SD—standard deviation; Min—minimum; Max—maximum; ME—median. NOTE: The relatively low ICU mortality observed in this cohort should be interpreted in light of the eligibility criteria. Inclusion did not require verbal communication or independent completion of the study instruments. Mechanically ventilated and sedated patients could be included when their level of arousal permitted a valid CAM-ICU-7 assessment. However, patients with deep sedation or coma who had a RASS score of minus 4 or minus 5 at the 12 h assessment were not included in the analytical cohort. Together with the exclusion of patients older than 80 years, patients with severe brain injury, patients with fever at the first assessment, and patients who died within the first 48 h, this may have selected a population with a relatively more favorable prognosis and may have contributed to the observed mortality rate of 4.49%.

**Table 2:** Distribution of CAM-ICU-7 severity categories at prespecified in-ICU assessment times.

	None		Mild/Moderate		Severe Delirium		N
	N	%	N	%	N	%	
CAM-ICU-7 12 h after admission	254	95.13%	11	4.12%	2	0.75%	267
CAM-ICU-7 2 days after admission	229	85.77%	29	10.86%	9	3.37%	267
CAM-ICU-7 4 days after admission	230	86.14%	21	7.87%	16	5.99%	267
CAM-ICU-7 1 week after admission	223	92.92%	7	2.92%	10	4.17%	240
CAM-ICU-7 2 weeks after admission	188	98.95%	2	1.05%	0	0.00%	190

N—number of patients; CAM-ICU-7—Confusion Assessment Method-Intensive Care Unit-7.

**Table 3:** Spearman rank correlations between delirium severity scores at consecutive in-ICU assessment times.

	CAM-ICU-7 12 h After Admission	CAM-ICU-7 2 Days After Admission	CAM-ICU-7 4 Days After Admission	CAM-ICU-7 1 Week After Admission	CAM-ICU-7 2 Weeks After Admission
CAM-ICU-7 12 h after admission	1.000	0.575	0.600	0.617	0.408
CAM-ICU-7 2 days after admission	0.575	1.000	0.983	0.795	0.277
CAM-ICU-7 4 days after admission	0.600	0.983	1.000	0.808	0.290
CAM-ICU-7 1 week after admission	0.617	0.795	0.808	1.000	0.352
CAM-ICU-7 2 weeks after admission	0.408	0.277	0.290	0.352	1.000

CAM-ICU-7—Confusion Assessment Method-Intensive Care Unit-7.

**Table 4:** Spearman rank correlations between inflammatory biomarkers and delirium severity across assessment times.

	CAM-ICU-7 12 h After Admission	CAM-ICU-7 2 Days After Admission	CAM-ICU-7 4 Days After Admission	CAM-ICU-7 1 Week After Admission	CAM-ICU-7 2 Weeks After Admission
CRP 12 h after admission	0.535	0.903	0.888	0.749	0.242
CRP 2 days after admission	-	0.898	0.885	0.740	0.241
CRP 4 days after admission	-	-	0.873	0.746	0.246
CRP 1 week after admission	-	-	-	0.719	0.253
CRP 2 weeks after admission	-	-	-	-	0.271
PCT 12 h after admission	0.304	0.251	0.275	0.420	0.401
PCT 2 days after admission	-	0.666	0.687	0.639	0.140
PCT 4 days after admission	-	-	0.711	0.682	0.301
PCT 1 week after admission	-	-	-	0.720	0.294
PCT 2 weeks after admission	-	-	-	-	0.329
IL-6 12 h after admission	0.583	0.595	0.586	0.555	0.351
IL-6 2 days after admission	-	0.735	0.721	0.692	0.298
IL-6 4 days after admission	-	-	0.694	0.719	0.282
IL-6 1 week after admission	-	-	-	0.727	0.287
IL-6 2 weeks after admission	-	-	-	-	0.312

CAM-ICU-7—Confusion Assessment Method-Intensive Care Unit-7; CRP—C-reactive protein; PCT—procalcitonin; IL-6—interleukin 6. Cells representing correlations in which the biomarker measurement occurred after the corresponding CAM-ICU-7 assessment (i.e., backward-looking, acausal comparisons) have been omitted from this table; only concurrent and forward-looking associations are shown.

**Table 5:** Spearman rank correlations between inflammatory biomarkers and ICU length of stay.

Variable	N	Spearman ρ	t(N − 2)	*p*-Value
CRP 12 h after admission	267	−0.030	−0.496	0.621
CRP 2 days after admission	267	−0.032	−0.527	0.599
CRP 4 days after admission	265	0.005	0.089	0.929
CRP 1 week after admission	247	0.128	2.017	0.045
CRP 2 weeks after admission	194	0.185	2.610	0.010
PCT 12 h after admission	267	0.151	2.489	0.013
PCT 2 days after admission	267	0.214	3.559	<0.001
PCT 4 days after admission	267	0.219	3.649	<0.001
PCT 1 week after admission	267	0.237	3.967	<0.001
PCT 2 weeks after admission	267	0.281	4.760	<0.001
IL-6 12 h after admission	267	0.036	0.586	0.558
IL-6 2 days after admission	266	0.077	1.255	0.211
IL-6 4 days after admission	265	0.109	1.771	0.078
IL-6 1 week after admission	242	0.148	2.314	0.021
IL-6 2 weeks after admission	188	0.186	2.586	0.010

N—number of patients; ρ—Spearman rank correlation coefficient; t(N − 2)—t statistic with N − 2 degrees of freedom; *p*—two-sided *p*-value.

## Data Availability

The datasets used and analyzed in this study are available from the corresponding author upon reasonable request.

## Supplementary Materials

The following supporting information can be downloaded at: https://www.mdpi.com/article/10.3390/jcm15156025/s1, Table S1: Distribution of CRP in the group of patients on individual days of hospitalization in the ICU; Table S2: Spearman rank correlations between CRP concentrations at consecutive ICU assessment times; Table S3: Distribution of ordinal PCT categories at prespecified assessment times relative to ICU admission; Table S4: Spearman rank correlations between ordinal PCT categories at prespecified assessment times relative to ICU admission; Table S5: Distribution of interleukin-6 concentration levels in the group of patients on individual days of hospitalization in the ICU; Table S6: Spearman rank correlations between IL-6 concentrations at consecutive ICU assessment times; Table S7: Apparent within-sample discrimination of baseline biomarkers for subsequent incident delirium; Table S8: Firth-penalized logistic regression for subsequent incident delirium; Table S9: Reason for hospitalization according to delirium status.

## Abbreviations

AUC	Area Under the Curve
aOR	adjusted Odds Ratio
CAM-ICU-7	Confusion Assessment Method for the Intensive Care Unit-7
CI	Confidence Interval
CRP	C-reactive protein
ELISA	Enzyme-Linked Immunosorbent Assay
ICU	Intensive Care Unit
IL-6	Interleukin-6
ME	Median
Min	Minimum
Max	Maximum
N	Number of patients
PCT	Procalcitonin
RASS	Richmond Agitation-Sedation Scale
ROC	Receiver Operating Characteristic
SD	Standard Deviation
