# Supplementary material for: Inflammatory Biomarkers and Delirium Severity in Critically Ill Adults: A Prospective Single-Center Cohort Study
Source: J Clin Med. 2026 Aug 3;15(15):6025. doi: 10.3390/jcm15156025 (PMC13466857; doi:10.3390/jcm15156025)
Supplement: Supplementary file 1 [file jcm-15-06025-s001.zip › jcm-4433598-supplementary.pdf]

SUPPLEMENTARY MATERIALS

Table S1. Distribution of CRP in the group of patients on individual days of hospitalization in the ICU.

	CRP ≤ 5 mg/L		CRP >5 mg/L		N
	N	%	N	%	
CRP 12 hours after admission [mg/L]	224	83.90%	43	16.10%	267
CRP 2 days after admission [mg/L]	224	83.90%	43	16.10%	267
CRP 4 days after admission [mg/L]	224	84.53%	41	15.47%	265
CRP 1 week after admission [mg/L]	216	87.45%	31	12.55%	247
CRP 2 weeks after admission [mg/L]	168	86.60%	26	13.40%	194

N – number of patients; CRP - C-reactive protein; mg/L - milligram/liter

Table S2. Spearman rank correlations between CRP concentrations at consecutive ICU assessment times.

	CRP 12 hours after admission	CRP 2 days after admission	CRP 4 days after admission	CRP 1 week after admission	CRP 2 weeks after admission
CRP 12 hours after admission [mg/L]	1.000	0.997	0.994	0.956	0.970
CRP 2 days after admission [mg/L]	0.997	1.000	0.996	0.960	0.971
CRP 4 days after admission [mg/L]	0.994	0.996	1.000	0.962	0.972
CRP 1 week after admission [mg/L]	0.956	0.960	0.962	1.000	0.997
CRP 2 weeks after admission [mg/L]	0.970	0.971	0.972	0.997	1.000

CRP - C-reactive protein; mg/L - milligram/liter

These near-unity serial correlations are driven largely by the shared floor distribution of CRP across time points (Supplementary Table S1) and should not be interpreted as evidence of tight biological tracking of CRP concentrations over time.

Table S3. Distribution of ordinal PCT categories at prespecified assessment times relative to ICU admission

	PCT ≤ 0.10 ng/mL		PCT 0.10 - 0.5 ng/mL		PCT > 0.5 ng/mL		
	N	%	N	%	N	%	N
12 h after admission	264	98.88%	3	1.12%	0	0.00%	267
2 days after admission	246	92.13%	3	1.12%	18	6.74%	267
4 days after admission	245	91.76%	0	0.00%	22	8.24%	267
1 week after admission	244	91.39%	1	0.37%	22	8.24%	267
2 weeks after admission	248	92.88%	1	0.37%	18	6.74%	267

N - number of patients; PCT - procalcitonin; ng/mL - nanogram/milliliter; % - percentage

Assessment times were defined relative to ICU admission. Later PCT samples could be obtained in the ICU, on another hospital ward after ICU discharge, or during a scheduled outpatient laboratory visit after hospital discharge. All categories were based on actual laboratory measurements obtained at the corresponding assessment time. No imputation, extrapolation, replacement, or last-observation-carried-forward procedure was used. PCT data were available for all 267 patients at each assessment time.

Table S4. Spearman rank correlations between ordinal PCT categories at prespecified assessment times relative to ICU admission

	PCT 12 h after admission	PCT 2 days after admission	PCT 4 days after admission	PCT 1 week after admission	PCT 2 weeks after admission
PCT 12 h after admission [ng/mL]	1.000	0.369	0.356	0.349	0.387
PCT 2 days after admission [ng/mL]	0.369	1.000	0.975	0.951	0.840
PCT 4 days after admission [ng/mL]	0.356	0.975	1.000	0.976	0.870
PCT 1 week after admission [ng/mL]	0.349	0.951	0.976	1.000	0.905
PCT 2 weeks after admission [ng/mL]	0.387	0.840	0.870	0.905	1.000

PCT – procalcitonin; ng/mL – nanogram/milliliter

PCT was analyzed as an ordinal variable with three categories: ≤ 0.10 ng/mL, 0.10 to 0.50 ng/mL, and > 0.50 ng/mL. Values are Spearman rank correlation coefficients.

These near-unity serial correlations are driven largely by the shared floor distribution of ordinal PCT categories across time points (Supplementary Table S3) and should not be interpreted as evidence of tight biological tracking of PCT concentrations over time.

Table S5. Distribution of interleukin-6 concentration levels in the group of patients on individual days of hospitalization in the ICU

	IL-6 ≤ 10 pg/mL		IL-6 > 10 pg/mL		N
	N	%	N	%	
IL-6 12 h after admission [pg/mL]	256	95.88%	11	4.12%	267
IL-6 2 days after admission [pg/mL]	241	90.60%	25	9.40%	266
IL-6 4 days after admission [pg/mL]	239	90.19%	26	9.81%	265
IL-6 1 week after admission [pg/mL]	215	88.84%	27	11.16%	242
IL-6 2 weeks after admission [pg/mL]	169	89.89%	19	10.11%	188

N - number of patients; IL-6 - interleukin-6; pg/mL - picograms per milliliter; % - percentage

Table S6. Spearman rank correlations between IL-6 concentrations at consecutive ICU assessment times

	IL-6 12 h after admission	IL-6 2 days after admission	IL-6 4 days after admission	IL-6 1 week after admission	IL-6 2 weeks after admission
IL-6 12 h after admission [pg/mL]	1.000	0.812	0.708	0.789	0.764
IL-6 2 days after admission [pg/mL]	0.812	1.000	0.913	0.921	0.868
IL-6 4 days after admission [pg/mL]	0.708	0.913	1.000	0.878	0.813
IL-6 1 week after admission [pg/mL]	0.789	0.921	0.878	1.000	0.978
IL-6 2 weeks after admission [pg/mL]	0.764	0.868	0.813	0.978	1.000

IL-6 – interleukin-6; pg/mL – picograms per milliliter

These near-unity serial correlations are driven largely by the shared floor distribution of IL-6 across time points (Supplementary Table S5) and should not be interpreted as evidence of tight biological tracking of IL-6 concentrations over time.

Table S7. Apparent within-sample discrimination of baseline biomarkers for subsequent incident delirium

Biomarker	N	Incident delirium cases	AUC	Bootstrap 95% CI	Exploratory Youden cutoff	Sensitivity, 95% CI	Specificity, 95% CI
CRP, mg/L	254	25	0.969	0.923 to 0.997	At least 8.0 mg/L	96.0% (79.6% to 99.9%)	97.4% (94.4% to 99.0%)
IL-6, pg/mL	254	25	0.653	0.569 to 0.750	At least 2.1 pg/mL	32.0% (14.9% to 53.5%)	98.7% (96.2% to 99.7%)

IL-6 - interleukin 6; pg/mL – picogram/milliliter; CRP - C-reactive protein; mg/L - milligram/liter

AUC values, cutoff values, sensitivity, and specificity were derived and evaluated in the same single-center cohort and represent apparent within-sample performance. The cutoff values were selected by maximizing the Youden index. No separate validation cohort was available, and calibration, optimism-corrected performance, net clinical benefit, and clinical utility were not assessed.

Table S8. Firth-penalized logistic regression for subsequent incident delirium

Model	Variable	Adjusted odds ratio	Profile-penalized 95% CI	Penalized likelihood ratio p-value
Separate CRP model	CRP, per 5 mg/L	10.30	4.66 to 29.04	<0.001
Separate CRP model	Age, per 10 years	1.02	0.68 to 1.62	0.929
Separate CRP model	Male sex versus female sex	1.07	0.34 to 3.68	0.907
Separate IL-6 model	IL-6, per 5 pg/mL	2.79	1.28 to 7.52	<0.001
Separate IL-6 model	Age, per 10 years	1.07	0.79 to 1.50	0.664
Separate IL-6 model	Male sex versus female sex	1.03	0.44 to 2.56	0.941
Joint biomarker model	CRP, per 5 mg/L	10.75	4.50 to 33.18	<0.001
Joint biomarker model	IL-6, per 5 pg/mL	0.81	0.42 to 2.04	0.510
Joint biomarker model	Age, per 10 years	1.00	0.67 to 1.59	0.995
Joint biomarker model	Male sex versus female sex	1.04	0.32 to 3.58	0.949

Adjusted odds ratios were estimated using Firth-penalized logistic regression. Confidence intervals were derived from the profile penalized likelihood, and two-sided p-values were obtained using penalized likelihood ratio tests.

Table S9. Reason for hospitalization according to delirium status

Reason for ICU admission	N	Delirium, n	Delirium, %
Circulatory failure	93	7	7.53%
Respiratory failure	43	12	27.91%
Acute renal failure	43	0	0.00%
Gastrointestinal bleeding	34	1	2.94%
Multi-organ trauma	24	3	12.50%
Hypothermia	19	13	68.42%
Acute pancreatitis	11	2	18.18%
Total	267	38	14.23%

N — number of patients; Delirium, n — patients with delirium; Delirium, % - percentage patients with delirium

This table is exploratory. Group sizes ranged from 11 to 93 patients; no formal significance testing was performed given the small numbers in several subgroups, and these associations should not be interpreted as adjusted or causal estimates.
